# Are Photosynthetic Characteristics and Energetic Cost Important Invasive Traits for Alien *Sonneratia* Species in South China?

**DOI:** 10.1371/journal.pone.0157169

**Published:** 2016-06-10

**Authors:** Feng-Lan Li, Qi-Jie Zan, Zheng-Yu Hu, Paul-K. S. Shin, Siu-Gin Cheung, Yuk-Shan Wong, Nora Fung-Yee Tam, An-Ping Lei

**Affiliations:** 1 College of Life Sciences, Shenzhen University, Shenzhen, China; 2 Futian-CityU Mangrove Research and Development Centre, City University of Hong Kong, Tat Chee Avenue, Kowloon, Hong Kong SAR, China; 3 Guangdong Neilingding Futian National Nature Reserve, Shenzhen, China; 4 Institute of Hydrobiology, Chinese Academy of Sciences, Wuhan, China; 5 Department of Biology and Chemistry, City University of Hong Kong, Tat Chee Avenue, Kowloon, Hong Kong SAR, China; 6 School of Science and Technology, Open University of Hong Kong, Homantin, Kowloon, Hong Kong SAR, China; University of A Coruña, SPAIN

## Abstract

A higher photosynthesis and lower energetic cost are recognized as important characteristics for invasive species, but whether these traits are also important for the ability of alien mangrove species to become invasive has seldom been reported. A microcosm study was conducted to compare the photosynthetic characteristics, energetic cost indices and other growth traits between two alien species (*Sonneratia apetala* and *S*. *caseolaris*) and four native mangrove species over four seasons in a subtropical mangrove nature reserve in Shenzhen, South China. The aim of the study was to evaluate the invasive potential of *Sonneratia* based on these physiological responses. The annual average net photosynthetic rate (P_n_), stomatal conductance (G_s_) and total carbon assimilation per unit leaf area (A_total_) of the two alien *Sonneratia* species were significantly higher than the values of the native mangroves. In contrast, the opposite results were obtained for the leaf construction cost (CC) per unit dry mass (CCM) and CC per unit area (CCA) values. The higher A_total_ and lower CC values resulted in a 72% higher photosynthetic energy-use efficiency (PEUE) for *Sonneratia* compared to native mangroves, leading to a higher relative growth rate (RGR) of the biomass and height of *Sonneratia* with the respective values being 51% and 119% higher than those of the native species. Higher photosynthetic indices for *Sonneratia* compared to native species were found in all seasons except winter, whereas lower CC values were found in all four seasons. The present findings reveal that alien *Sonneratia* species may adapt well and become invasive in subtropical mangrove wetlands in Shenzhen due to their higher photosynthetic characteristics coupled with lower costs in energy use, leading to a higher PEUE. The comparison of these physiological responses between *S*. *apetala* and *S*. *caseolaris* reveal that the former species is more invasive than the latter one, thus requiring more attention in future.

## Introduction

Biological invasions are one of the most important threats affecting biodiversity, species composition, and the structure and function of ecosystems, and have caused significant global economic losses [1,2]. Many attempts have been made to identify the properties of species that predispose them to becoming invasive [3], but few generalities have emerged that would allow us to predict which introduced species may become aggressive invaders [4]. Photosynthesis, which converts solar energy into carbohydrate molecules, is an important physiological process that supplies energy and photoassimilates in plants. A higher photosynthetic rate (P_n_) than that of native species has been recognized as a significant characteristic of invasive species [5–8]. Previous studies have shown that successful invasive species have other morphological and physiological traits that increase their light capture and utilization efficiency [5]. Invasive plants can be successful by maximizing photosynthesis and growth [6,7], and the success of many rapidly growing, often invasive species has been attributed to higher photosynthetic nitrogen-use efficiency (PNUE) compared to slower growing native species [9–16]. Leaf construction cost (CC), a quantifiable index of the energy demand for biomass production [17,18], is defined as the amount of glucose required to provide carbon skeletons, reductants such as NADPH, and energy (e.g., ATP) for the synthesis of organic compounds [19]. In contrast to the P_n_ and PNUE, a lower CC generally indicates a higher relative growth rate (RGR), and even small differences in the CC can lead to large differences in the growth rate [18]. Thus, a low CC with efficient energy utilization is advantageous for plant growth and in turn may increase the abundance and spread of a species [20]. Song et al. [21] compared the photosynthetic ability, energetic cost index and other growth traits between alien and native species and found that these physiological factors influencing invasions were important measures for better prediction and control of potentially invasive alien species.

Futian Nature Reserve (22°31' N, 114°05' E) is located in Shenzhen city and is an important wetland in Southern China opposite of the Mai Po Nature Reserve, which is a well-known mangrove wetland in Hong Kong SAR and part of the RAMSAR. The dominant native mangrove species are *Avicennia marina* (Am), *Kandelia obovata* (Ko), *Aegiceras corniculatum* (Ac) and *Bruguiera gymnorrhiza* (Bg) [22]. In the late 1990s, a number of alien mangrove species, including *Sonneratia caseolaris* (Sc), *S*. *apetala* (Sa), *Bruguiera sexangula* (Bs), *Rhizophora stylosa* (Rs) and *Avicennia marina* var. australasica (AmA), were purposefully introduced to the lower tidal mudflat areas of the reserve for reforestation projects [22,23]. Since their introduction, the two *Sonneratia* species have grown very quickly, produced many fruits and seeds, and spread to other regions in the reserve as well as to the Mai Po RAMSAR in Hong Kong; in contrast, the other alien mangroves grew slowly and were restricted to the planted areas [23–26]. The governments of both Shenzhen and Hong Kong have spent resources to eradicate the *Sonneratia* seedlings and mature adults that have colonized outside the planted areas since the mid-2000s; however, the removal is often expensive, labor intensive and ineffective.

*Sonneratia* has a fast growth rate and high tolerance of environmental stresses, which is why these species have been used for mangrove restoration projects [27,28]. However, the invasiveness of *Sonneratia* species and the possibility of replacing native mangrove species have been subjects of debate in China, and there are disagreements on whether *Sonneratia* should be planted [28,29]. Some researchers concluded that the competitive advantages, such as high productivity [24,30,31], high rates of seed dispersal and germination [23,26], and the invasive potential of the two *Sonneratia* species, should not be neglected. We also found that the two *Sonneratia* species had a higher specific leaf area (SLA) and a lower leaf CC than the native mangrove species in Shenzhen, indicating their competitiveness and invasive potential [32]. Chen et al. [33] reported that the two introduced *Sonneratia* had comparable photosynthetic rates but no higher carbon assimilation than native mangrove species in late fall (November) in Shenzhen. Other studies showed that the P_n_ of *Sonneratia* was significantly higher than that of native mangroves in summer [34–36]. Our previous studies in 2011 [32] mainly focused on the CC between adult alien and native mangrove plants in only one season (summer). To make a more meaningful comparison between alien and native species, seasonal variations in growth should be systematically explored. Additionally, most previous studies primarily focused on photosynthetic traits, whereas few studies investigated the energy cost of mangroves even though this trait is also important for the evaluation of the invasive potential of an alien plant. To the best of our knowledge, no study has combined the photosynthetic characteristic and CC of *Sonneratia* to assess its invasive potential. The present study investigated both the photosynthetic characteristics and energetic cost of the seedlings of *Sonneratia* and native species in four seasons throughout a whole year, aiming to provide a more comprehensive evaluation on the invasive potential of the alien *Sonneratia* species. In addition, young seedlings instead of mature adults were used in this study to better understand the invasiveness of *Sonneratia* in an early age, as no evaluation on their invasiveness at such early age were reported.

The present field microcosm study has the following aims: (i) to compare the photosynthetic characteristics, leaf CC and other growth traits of the seedlings of two *Sonneratia* and four native mangrove species across four seasons and (ii) to evaluate the invasive potential of the alien *Sonneratia* in South China. The two alien *Sonneratia* were Sc and Sa, and the four native species (Bg, Ko, Ac and Am) were the dominant native mangrove species in Shenzhen. The photosynthetic characteristics included the net photosynthetic rate (P_n_), stomatal conductance (G_s_), rate of transpiration (E), intercellular CO_2_ concentration (C_i_) and total carbon assimilation per unit leaf area (A_total_). Leaf CC was presented in terms of CC per unit dry mass (CCM) and CC per unit area (CCA). Other growth traits were biomass, height, ground diameter, and RGR for biomass (RGR_Biomass_), height (RGR_Height_) and ground diameter (RGR_Diameter_).

## Materials and Methods

### Study site and cultivation of mangrove seedlings

The study was performed in the Futian Mangrove Nature Reserve (22°31' N, 114°05' E) located in an estuary of the Zhujiang River in Shenzhen, Guangdong Province, China. The study site is characterized by a subtropical monsoonal climate with an annual precipitation of 1927 mm and a rainy season from May to September [37]. The monthly change in precipitation was around 26.3–553.8 mm. The mean annual air temperature is 22°C, with a low mean monthly temperature of 14°C (in January) and a high mean monthly temperature of 28°C (in July) [37]. The monthly changes in air temperature in summer and winter seasons were 27.8–28.9°C and 15.9–18.4°C, respectively. The mean monthly insolation is 450 j·m^-2^, with the highest insolation in August (590 j·m^-2^) and the lowest in February (322 j·m^-2^) [37]. The mean duration of light is 6.1 h·day^-1^, with the minimum and maximum hours occurring in February (4.2 h·day^-1^) and August (7.8 h·day^-1^), respectively [37]. The study site area has the features of non-regular, semi-diurnal tides and the spring tidal range is about 2.8 m [37].

The seedlings of the four native mangrove species were germinated from seeds or propagules harvested from the nursery garden of the Futian Mangrove Nature Reserve prior to the study, while that of the two *Sonneratia* species were collected from the monoculture forest of the reserve. To ensure that all seedlings had comparable morphological features and sizes, all native species were 12 month-old. However, as the aliens, *Sonneratia*, grew faster than the native species, the 12 month-old seedlings were too big with extensive roots to be transplanted, leading to a high death rate of the transplanted seedlings. Therefore, younger seedlings of *Sonneratia* (3- to 4-month old) with morphology and size similar to the native mangrove seedlings (12-month old) were used (Table 1).

**Table 1:** Information on mangrove seedlings transplanted to pots on 12^th^ July, 2010.

Species	Family name	Full name	Source of seedling	Age of seedlings (months)	Seedling height (cm)	Ground diameter (cm)	Moisture content (%)	Fresh biomass (g)	Dry biomass (g)
**Alien *Sonneratia* species**	Sonneratiaceae	*Sonneratia caseolaris*	Forest of *S*. *caseolaris*	3–4	17.7±1.8	0.90±0.05	86.0±0.6	253.0±13.9	35.2±0.6
	Sonneratiaceae	*S*. *apetala*	Forest of *S*. *apetala*	3–4	17.9±2.1	0.50±0.02	78.2±0.3	156.1±13.3	33.9±2.5
**Native**	Rhizophoraceae	*Bruguiera gymnorrhiza*	Nursery garden	12	34.5±3.5	0.36±0.02	76.3±0.2	87.6±1.3	20.8±0.2
	Rhizophoraceae	*Kandelia obovata*	Nursery garden	12	35.6±3.1	0.27±0.01	79.1±0.1	82.2±6.2	17.2±1.3
	Myrsinaceae	*Aegiceras corniculatum*	Nursery garden	12	30.2±2.6	0.41±0.03	75.4±0.6	149.2±3.4	36.7±1.8
	Verbenaceae	*Avicennia marina*	Nursery garden	12	41.4±3.8	0.40±0.02	77.0±0.8	94.9±17.7	21.8±3.9

Mean and standard error of replicates are shown for the height, diameter, moisture and biomass.

At the beginning of the experiment, uniformly sized seedlings of the same species were transplanted into pots filled with mangrove soil collected from the reserve on 12^th^ July 2010. Each pot had a dimension of 45 cm in height and approximately 45 cm in diameter and contained one transplanted seedling. Prior to transplanting, the seedling was gently washed with tap water to remove mud and wiped with tissue paper. The height, ground diameter, moisture content, and fresh and dry initial biomass of the seedlings were determined and are summarized in Table 1. All pots with a tray at the bottom of each were placed on the open ground at the landward edge of the mangrove forest in the reserve, and the seedlings were grown for 18 months prior to the first measurement on 12^th^ January 2012. During high tides, tidal water was flooded to the tray but not to the surface of the pot. The bottom soil could be wetted from below. To have sufficient moisture for plant growth, the pots were watered daily (at 18:00 h) by the tidal water collected from a nearby drain connecting the sea, about 3 litre tidal water per pot, except rainy days, to simulate the natural condition of the mangrove forest. At the end of the 18 months growth period, four consecutive samplings were carried out, i.e., in January, April, July and October, representing the winter, spring, summer and fall seasons, respectively. For each species, a total of 12 pots were prepared, with three pots randomly sampled in each season. In every sampling season, the photosynthesis of each plant in the three pots were measured *in situ*, and these plants were harvested for the determination of the morphological index, CC and growth traits, with triplicates for each species.

### Measurement of photosynthetic indices and estimation of energy gain

The photosynthetic parameters of the fully expanded mature leaves (i.e., the 2^nd^ or 3^rd^ pair of leaves from the tip of the stem) of each plant were measured *in situ* using a Li-6400 portable photosynthesis system with a standard 6 cm^2^ leaf chamber (Li-6400, Li-Cor, USA). The measurements were performed on sunny days on January 12^th^ (winter), April 16^th^ (spring), July 19^th^ (summer) and October 16^th^ (fall) in 2012. Conditions inside the leaf chamber during the gas exchange measurements were controlled as follows: Irradiance was provided by an integrated red-blue light emitting at 1500 μmol m^-2^s^-1^, and the leaf temperature was automatically controlled at 28°C. The photosynthetic indices, including the net photosynthetic rate (P_n_, μmol m^-2^s^-1^), stomatal conductance (G_s_, mmol m^-2^s^-1^), transpiration rate (E, mmol m^-2^s^-1^) and intercellular CO_2_ concentration (C_i_, μmol mol^-1^), were recorded after a steady state was reached (coefficient of variation < 1%) for each measurement. For each plant in a pot, the photosynthetic indices of five leaves were measured, and the average of the five measurements was calculated and taken as one replicate of that species. On each measurement date, the diurnal photosynthetic indices of each mangrove species were measured from 08:00 to 18:00 at 2 h intervals. The average daily P_n_ was determined by scaling the six instantaneous net P_n_ on the same day measured from each individual mangrove seedlings.

The energy gain during the one year sampling period (equivalent to the total carbon fixation per unit leaf area) was estimated using the measured photosynthetic rate (P_n_) from four different seasons as mentioned above. Because the daily fluctuation of the photosynthetic rate within the same season was relatively small, the average daily P_n_ was considered the mean P_n_ in a given season, and daily carbon fixation was calculated by scaling the daily average P_n_ for a 16 h photoperiod. The total carbon gain per unit leaf area (A_total_) in terms of mol CO_2_ m^-2^ was calculated by summing the daily carbon gain across the different seasons throughout the year [21,38,39].

### Measurement of morphological growth traits

On each sampling date, the harvested plant was divided into leaves, stems and roots, washed with tap water and dried with absorbent paper. The biomass of each plant component, the height and ground diameter of the main stem, and the elemental composition and CC of the mature leaves were determined. The RGR_Biomass_, RGR_Height_ and RGR_Diameter_ were calculated according to the following equations:

RGRBiomass=(lnW2−lnW1)t

RGRHeight=(lnH2−lnH1)t

RGRDiameter=(lnD2−lnD1)t

where W_1_, H_1_ and D_1_ were the initial biomass, height and ground diameter, respectively, W_2_, H_2_ and D_2_ were the respective values at the next harvest, and t was the interval time (days) between the two harvests.

### Estimation of the leaf construction cost (CC)

On each sampling date, approximately 30 fully expanded mature leaves were randomly selected from each individual plant. The leaf construction cost (CCM, equivalent to grams glucose per gram dry mass, g(glucose)g^-1^) was estimated according to the method described by Williams et al. [19] as follows:

CCM=[(0.06968×ΔHc−0.065)(1−Ash)+7.5(kN/14.0067)]EG

where *k* was the oxidation state of the N substrate (+5 for nitrate or -3 for ammonium). EG was the growth efficiency and was estimated across species to be 0.87 according to Penning de Vries et al. [40]. The leaf CC based on area (CCA, equivalent to grams glucose per square meter, g(glucose) m^-2^) was calculated by the formula:

CCA=CCMSLA

The parameters in the above formulas (i.e., ΔH_c_, Ash, N and SLA) were determined and calculated according to our previous studies [32].

### Estimation of photosynthetic energy-use efficiency (PEUE) and photosynthetic nitrogen-use efficiency (PNUE)

The photosynthetic energy-use efficiency (PEUE, mol CO_2_ g^-1^ glucose) was calculated by the ratio of A_total_ to the leaf CCA according to Nagel et al. [39]. The photosynthetic nitrogen-use efficiency (PNUE, mol CO_2_ g^-1^ N) was calculated by the ratio of A_total_ to the N concentration based on the unit area [N] [21].

### Data analyses

The photosynthetic index, leaf CC, PEUE, PNUE and RGR among the six species and four seasons were analyzed using a parametric two-way analysis of variance (ANOVA) with species (n = 6) and seasons (n = 4) as the sources of variation followed by a Student-Newman-Keuls (S-N-K) test for multiple comparisons. The differences between alien *Sonneratia* and native mangrove groups were tested by the independent samples T-test. All data fulfilled the assumptions of the parametric test, and no data transformation was needed. The statistical analyses were performed using commercial SPSS 17.0 software (SPSS Inc., USA).

### Ethics Statement

Field permit issued by the Futian Mangrove Nature Reserve in Shenzhen, Guangdong Province, China, allowed us to collect the mangrove seedlings and perform the study in this site. They also confirmed that our study did not involve any endangered or protected species.

## Results

### Photosynthetic indices

**Fig 1:**
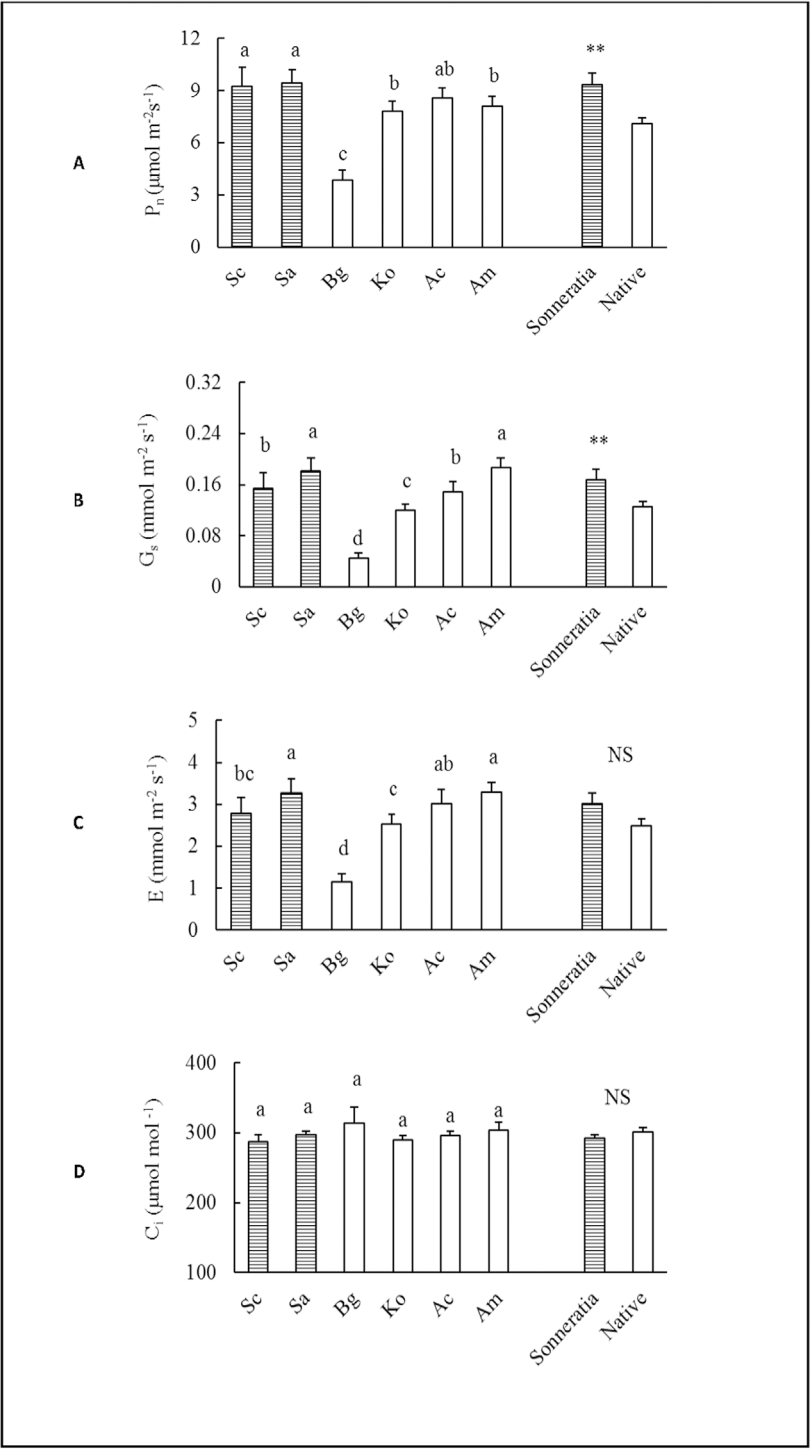
**Comparisons of the annual average (A) net photosynthetic rate (P**_**n**_**), (B) stomatal conductance (G**_**s**_**), (C) rate of transpiration (E) and (D) intercellular CO**_**2**_ **concentration (C**_**i**_**) between alien *Sonneratia* and native mangrove species.** Fig 1 legend: Error bars represent 1 SE. Different letters indicate significant differences at p ≤ 0.05 among the six species according to two-way ANOVA; ** indicates a significant difference between alien *Sonneratia* species and native mangroves at p ≤ 0.01, NS indicates not significant at p ≤ 0.05 according to the independent samples T-test.

**Fig 2:**
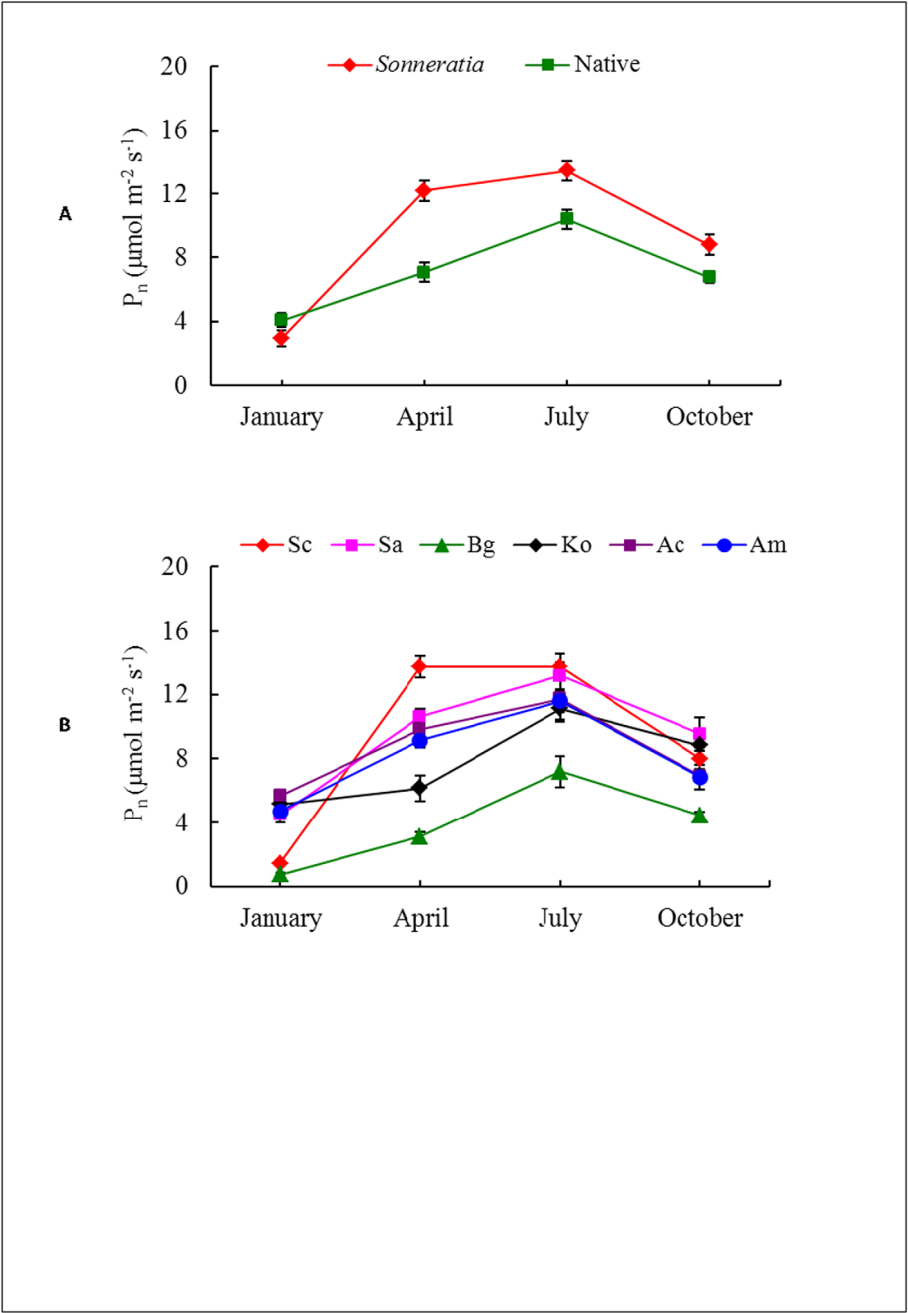
Seasonal dynamics of the net photosynthetic rate (P_n_) of alien *Sonneratia* and native mangrove species. Fig 2 legend: Error bars represent ±1 SE.

The annual averages of P_n_ and G_s_ of the alien *Sonneratia* were significantly higher than those of the native mangrove species according to the independent samples T-test (t_Pn_ = 3.35, p ≤ 0.01; t_Gs_ = 2.67, p ≤ 0.01) (S1 Table), with increases of 32% and 34%, respectively (Fig 1**A** and 1**B**). The higher P_n_ of the *Sonneratia* species resulted in a 32% higher A_total_ compared to the native mangroves (t_Atotal_ = 3.34, p ≤ 0.01) (S1 Table); the A_total_ values of the alien and native species were 49.33 and 37.39 mol CO_2_ m^-2^, respectively. However, there were no significant differences in E and C_i_ between the alien *Sonneratia* and native mangrove species, with t_Ci_ = -0.80 and t_E_ = 1.87 (p ≥ 0.05) (Fig 1**C** and 1**D**, S1 Table). Both species and seasonal effects on all determined photosynthetic indices were significant according to the two-way ANOVA with the exception of the species effects on C_i_ (Table 2, Fig 2A and 2B). Among the six mangrove species, Sa and Sc had the highest P_n_. However, the highest G_s_ and E were found in Sa and Am (Fig 1**A**–1**D**). Among the four native species, Ac had the highest P_n_, and Bg had the lowest values in all seasons (Fig 1**A**). For the two *Sonneratia* species, no significant differences in annual average P_n_ and A_total_ were found. The P_n_ values of Sa and Sc were 9.45 and 9.23 μmol m^-2^s^-1^, respectively, whereas the respective A_total_ values were 49.88 and 48.78 mol CO_2_ m^-2^. The photosynthetic indices were also significantly affected by the season, with the P_n_ of both alien and native mangroves increasing from winter (January) to spring (April), reaching a maximum in summer (July) and declining in fall (October). Generally, seasonal variations were more obvious in the alien *Sonneratia* than in the native mangrove species (Fig 2**A**). Among the six mangrove species, the P_n_ of Sc in the winter was significantly lower than that of the native mangrove species with the exception of Bg (Fig 2**B**).

**Table 2:** F values of the two-way ANOVA test showing the effects of species and seasons and their interactions on the photosynthetic characteristics, energetic cost and growth traits of mangrove plants.

Items	F-value		
	Species (n = 6)	Season (n = 4)	Species×Season
**P_n_**	38.35***	142.75***	7.61***
**G_s_**	41.65***	95.90***	10.56***
**E**	37.97***	171.51***	6.31***
**C_i_**	1.60^NS^	44.53***	6.71***
**A_total_**	38.31***	145.47***	7.62***
**CCM**	135.01***	51.51***	3.93**
**CCA**	27.86***	10.72***	5.16***
**PEUE**	20.70***	32.78***	4.57***
**PNUE**	24.05***	80.47***	4.09***
**RGR_Biomass_**	4.36**	3.85**	1.47^NS^
**RGR_Height_**	4.48**	4.64**	1.93*
**RGR_Diameter_**	1.73^ns^	9.93***	1.61^NS^

P_n_ = net photosynthetic rate, G_s_ = stomatal conductance, E = rate of transpiration, C_i_ = intercellular CO_2_ concentration, A_total_ = total carbon assimilation per unit leaf area, CCM = leaf construction cost per unit dry mass, CCA = leaf construction cost per unit area, PEUE = photosynthetic energy-use efficiency, PNUE = photosynthetic nitrogen-use efficiency. RGR_Biomass_, RGR_Height_ and RGR_Diameter_ are the relative growth rates of biomass, height and ground diameter of six mangroves, respectively. Levels of significance are shown as

* *p ≤* 0.05

** *p ≤* 0.01

*** *p ≤* 0.001

and NS for not significant at *p ≤* 0.05.

### Leaf CC

**Fig 3:**
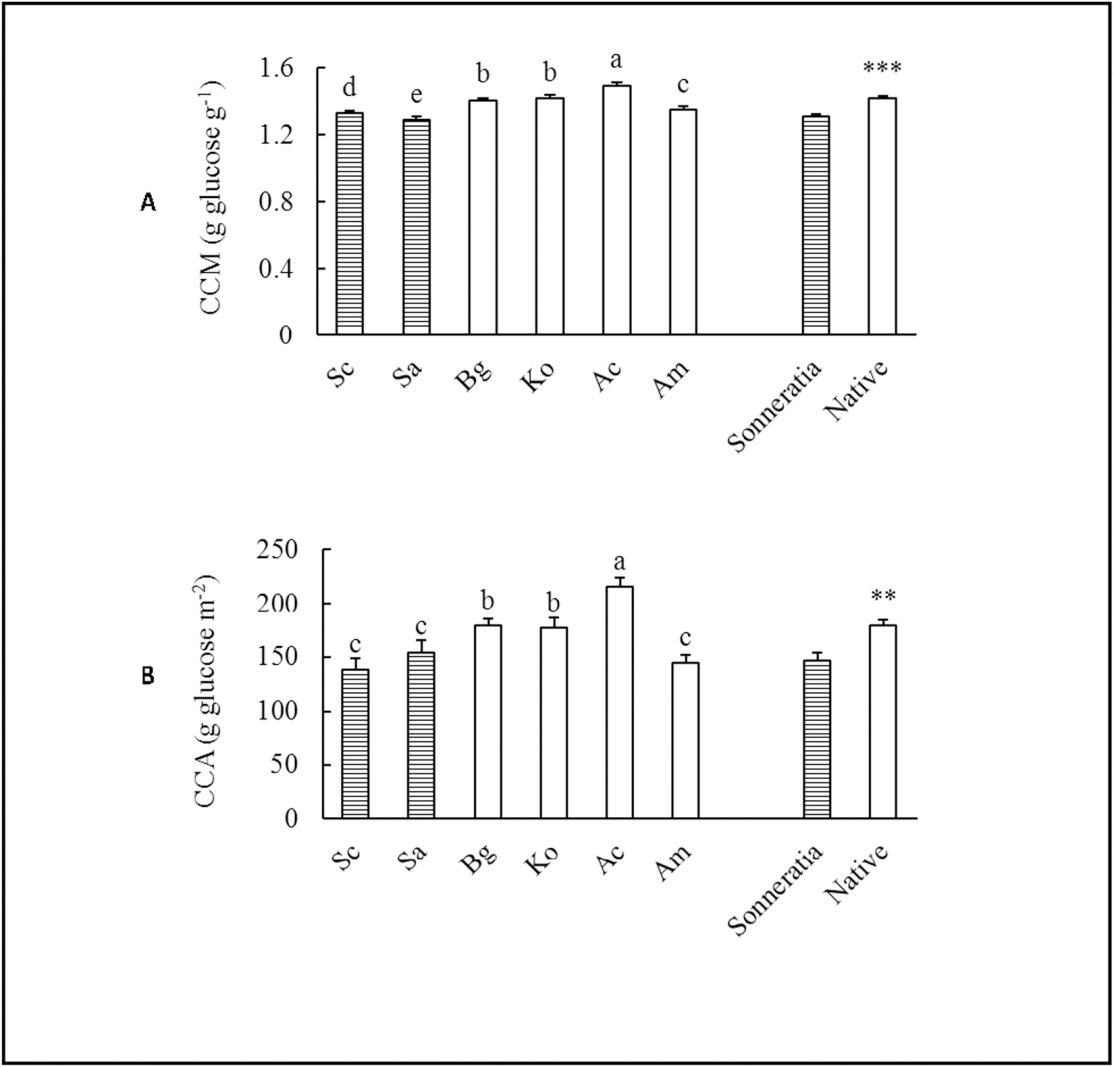
**Comparisons of the annual average (A) leaf CCM and (B) CCA between alien *Sonneratia* and native mangrove species.** Fig 3 legend: Error bars represent 1 SE. Different letters indicate significant differences at p ≤ 0.05 among the six species according to two-way ANOVA; ** and *** indicate significant differences between alien *Sonneratia* and native mangrove species at p ≤ 0.01 and p ≤ 0.001 according to the independent samples T-tests, respectively.

**Fig 4:**
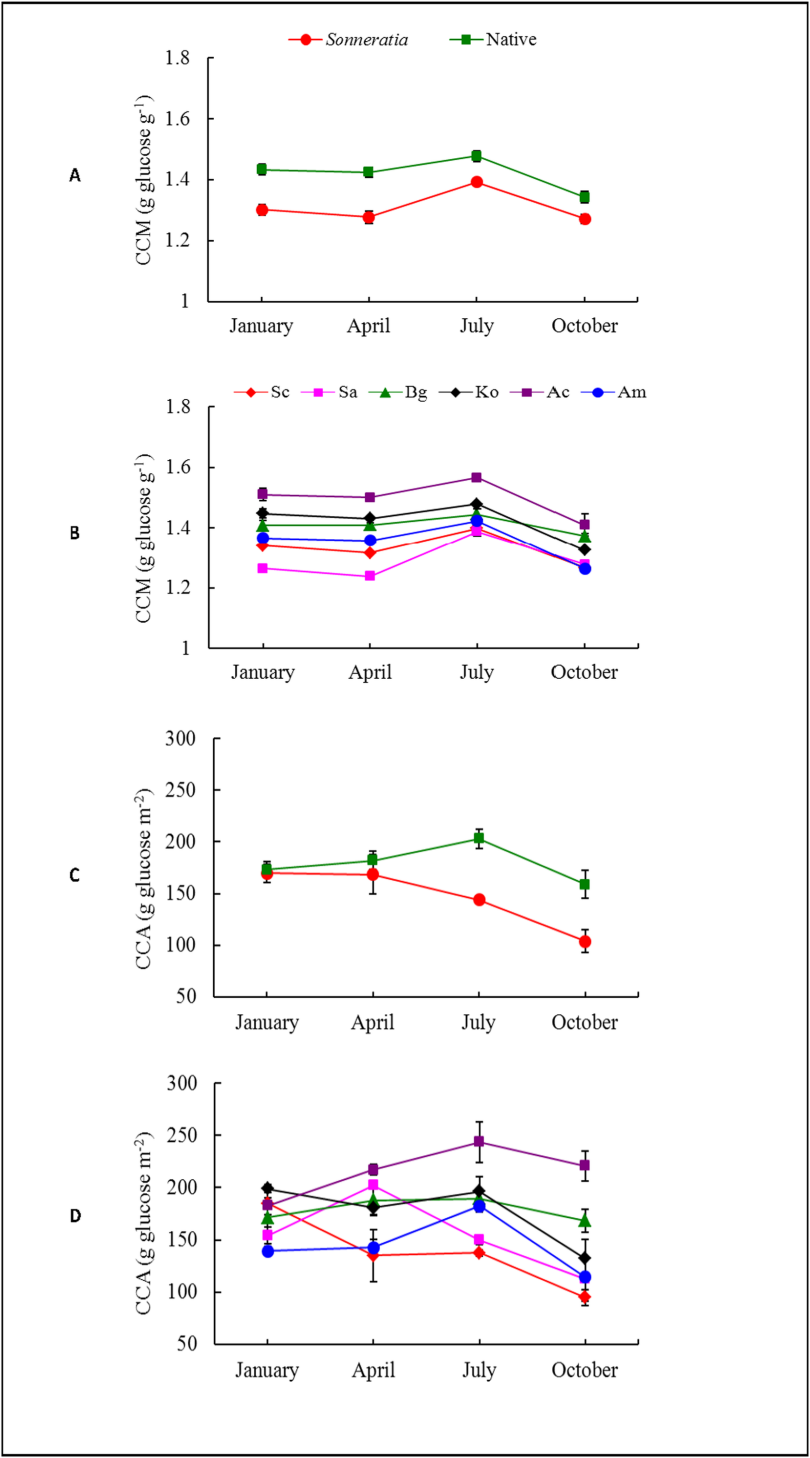
**Seasonal dynamics of (A, B) CCM and (C, D) CCA of alien *Sonneratia* and native mangrove species.** Fig 4 legend: Error bars represent ±1 SE.

In contrast to P_n_, the annual averages of both leaf CCM and CCA of the alien *Sonneratia* were significantly lower than those of the native mangrove species (t_CCM_ = -6.11, p ≤ 0.001; t_CCA_ = -3.48, p ≤ 0.01) (S1 Table), with differences of 7.68% and 18.39%, respectively (Fig 3**A** and 3**B**). The effects of species, seasons, and their interactions on both CCM and CCA were significant according to the two-way ANOVA (Table 2, Fig 4**A**–4**D**). Large significant differences in leaf CC were found among the six mangrove species. Sa had the lowest CCM, and Sc had the lowest CCA, whereas Ac had the highest CCM and CCA (Fig 3**A** and 3**B**). The seasonal CC patterns were very different from those of P_n_. The leaf CCM values of *Sonneratia* were relatively low and were significantly lower than those of the native species in all four seasons (t_winter_ = -4.74, p ≤ 0.001; t_spring_ = -5.58, p ≤ 0.001; t_summer_ = -3.40, p ≤ 0.01; t_fall_ = -2.53, p ≤ 0.05) (Fig 4**A** and 4**B**, S1 Table), whereas the differences in CCA between *Sonneratia* and native species were only found in summer and fall but not in winter and spring (t_winter_ = -0.28, p ≥ 0.05; t_spring_ = -0.75, p ≥ 0.05; t_summer_ = -4.44, p ≤ 0.001; t_fall_ = -2.66, p ≤ 0.05) (Fig 4**C** and 4**D**, S1 Table). The CC of native mangroves followed seasonal patterns similar to those observed for *Sonneratia*, with the highest values in summer (Fig 4**A**–4**D**).

### PEUE and PNUE

**Fig 5:**
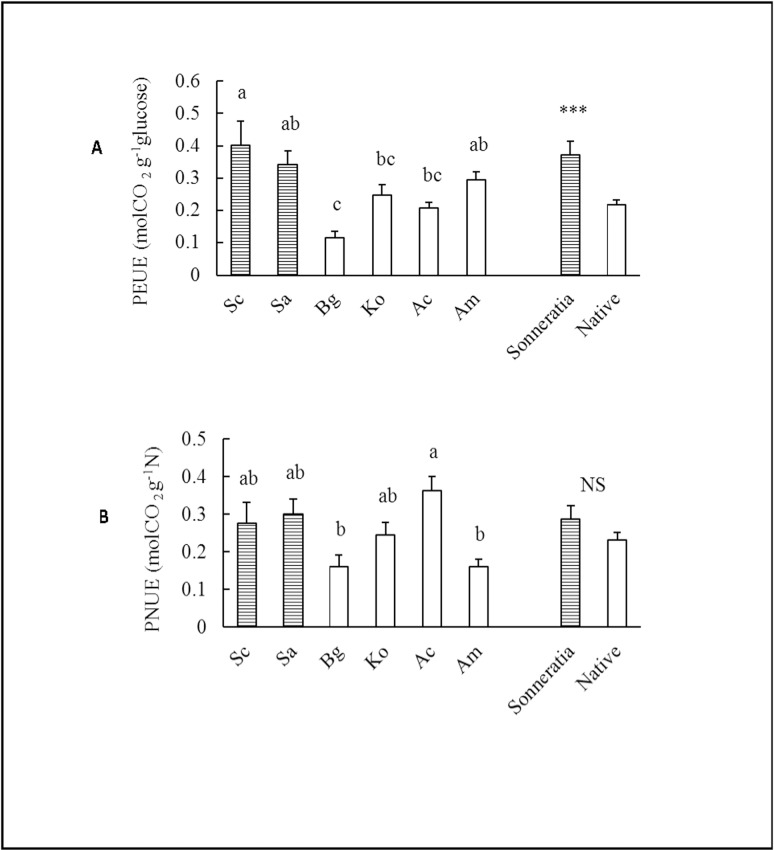
**Comparisons of the annual average (A) PEUE and (B) PNUE between alien *Sonneratia* and native mangrove species.** Fig 5 legend: Error bars represent 1 SE. Different letters indicate significant differences at p ≤ 0.05 among the six species according to two-way ANOVA; *** indicates a significant difference between alien *Sonneratia* and native mangrove species at p ≤ 0.001, and NS indicates not significant at p ≤ 0.05 according to the independent samples T-test.

**Fig 6:**
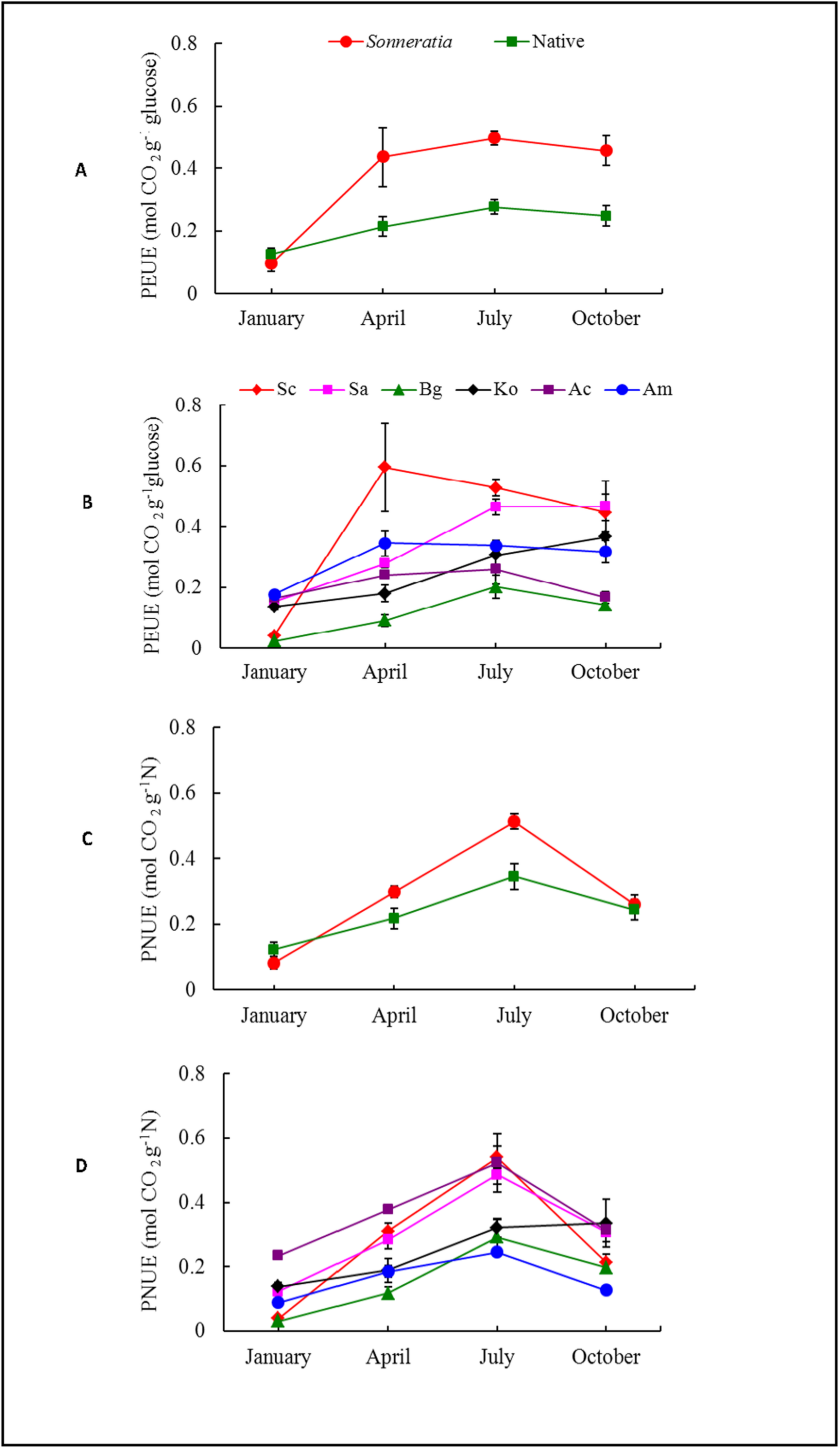
**Seasonal dynamics of (A, B) PEUE and (C, D) PNUE of alien *Sonneratia* and native mangrove species.** Fig 6 legend: Error bars represent ±1 SE.

Due to their higher A_total_ and lower CC, the *Sonneratia* species had a 72% higher annual average PEUE than the native mangroves (t_PEUE_ = 4.22, p ≤ 0.001) (Fig 5**A**, S1 Table). Significant differences in PEUE were found among the six species, with Sc exhibiting the highest value and Bg the lowest (Fig 5**A**). The differences between the two *Sonneratia* species were not significant. Among the four native species, the PEUE of Am was the highest and that of Bg was the lowest (Fig 5**A**). The species and seasonal effects and their interactions on PEUEs were also significant (Table 2, Fig 6**A** and 6**B**). The seasonal pattern of PEUEs of *Sonneratia* was similar to that of P_n_, with the lowest value in winter, an increase in spring and high levels in the summer and fall (Fig 6**A**). The PEUEs of *Sonneratia* were significantly higher than those of the native species during the four seasons except winter (t_winter_ = -0.90, p ≥ 0.05; t_spring_ = 2.28, p ≤ 0.05; t_summer_ = 6.11, p ≤ 0.001, t_fall_ = 3.67, p ≤ 0.01) (Fig 6**A**, S1 Table). The seasonal variations in PEUE of the native species were less obvious compared to those of *Sonneratia* (Fig 6**A** and 6**B**). Similar to P_n_, the PEUE of Sc in winter was significantly lower than the values of the native mangrove species with the exception of Bg (Fig 6**B**).

There were no significant differences in the annual average PNUE between *Sonneratia* and the native mangrove species (t_PNUE_ = 1.55, p ≥ 0.05) (Fig 5**B**, S1 Table); the differences were also not significant during the four seasons except summer (t_winter_ = -1.18, p ≥ 0.05; t_spring_ = 1.75, p ≥ 0.05; t_summer_ = 2.84, p ≤ 0.05; t_fall_ = 0.35, p ≥ 0.05) **(**Fig 6**C**, S1 Table**).** Among the six species, Ac had the highest PNUE, while the lowest values were found in Am and Bg. Ac may have had the highest PNUE due to its low leaf nitrogen concentration. Among the four seasons, all six mangroves had the highest PNUE in summer and the lowest in winter (Fig 6**D**).

### Other growth traits

**Fig 7:**
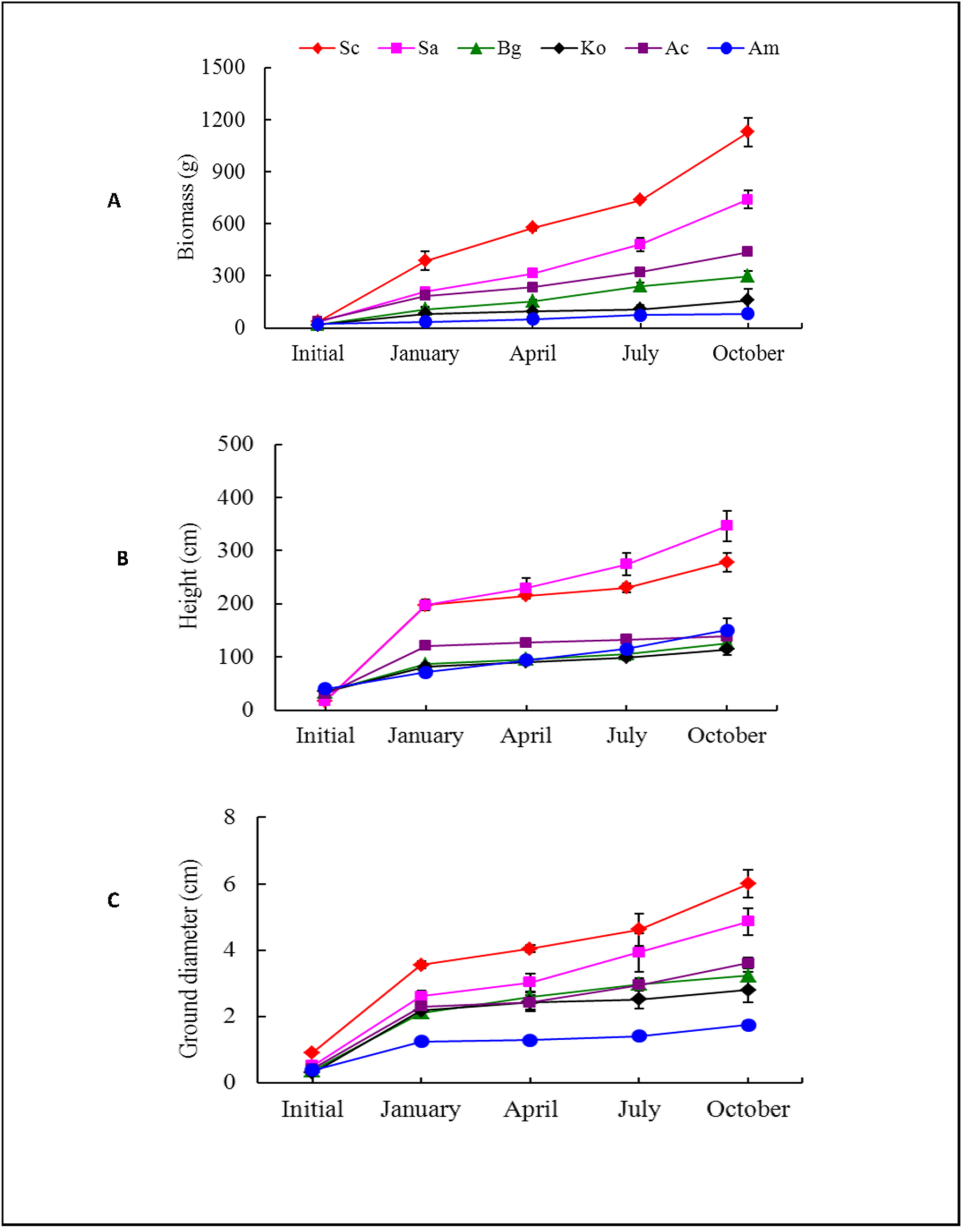
**Dynamics of (A) biomass, (B) height and (C) ground diameter of six mangrove species at the beginning of the experiment and in the four consecutive seasons.** Fig 7 legend: Error bars represent ± 1 SE.

**Fig 8:**
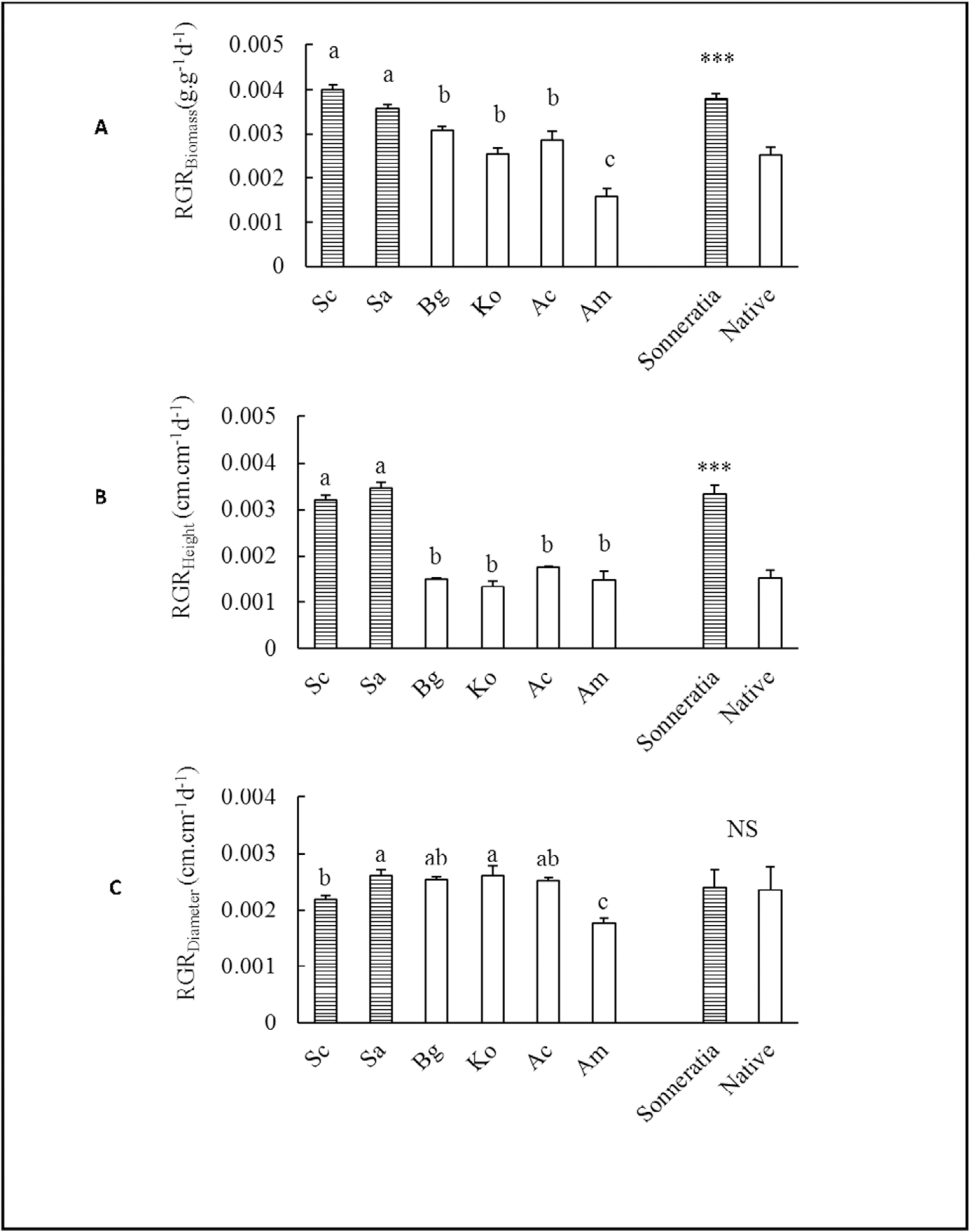
**Comparisons of (A) RGR**_**Biomass**_**, (B) RGR**_**Height**_ **and (C) RGR**_**Diameter**_ **between alien *Sonneratia* and native mangrove species.** Fig 8 legend: Error bars represent 1 SE. Different letters indicate significant differences at p ≤ 0.05 among the six species according to two-way ANOVA; *** indicates a significant difference between alien *Sonneratia* and native mangrove species at p ≤ 0.001, and NS indicates no significant difference at p ≤ 0.05 according to the independent samples T-test.

The growth of *Sonneratia*, including the biomass, height and ground diameter growth, was significantly greater than that of the native mangrove species throughout the entire year, and the differences increased over time (Fig 7**A**–7**C**). Among the two *Sonneratia* species, the biomass and ground diameters of Sc were significantly higher than those of Sa in all four seasons, but the heights of Sc in summer and fall were significantly lower than those of Sa (Fig 7). At the end of the experiment, Sc had the greatest biomass and ground diameter, whereas Sa was the tallest species. In contrast, the pioneer native mangrove species Am, which occupies a tidal position similar to *Sonneratia*, exhibited the lowest growth rate (Figs 7 and 8). Due to the greater growth of *Sonneratia*, the RGR_biomass_ and RGR_height_ were 51% and 119% higher than the values of the native mangroves, respectively (Fig 8**A**–8**C**).

## Discussion

Invasive plants have been reported to often have higher gas exchange rates than indigenous species [5–7,41], and higher leaf gas exchange rates are often attributed to the competitive nature of invasive species in non-native habitats [42]. A high rate of net carbon gain indicates high productivity and is a trait of many invasive species [15,43]. In the present study, the annual averages of P_n_, G_s_ and A_total_ of alien *Sonneratia* were 32%, 34% and 32% higher than those of the native mangrove species, respectively. The differences were most substantial in the spring and summer seasons (Fig 2), indicating a higher competitive ability and thus invasive potential in the two seasons.

CC is a quantifiable measure of energy demand for biomass production. A low CC is hypothesized to give advantages to the growth of an alien plant because it is associated with a high RGR and thus increases the invasive potential. A small difference in CC has been reported to lead to a substantial difference in the growth rate and an increase in the invasive potential [17,18]. The lower CC but higher A_total_ of the alien species indicated that this species could assimilate the amount of energy needed to construct tissues through photosynthesis in a relatively short time with more energy left over for growth and seed production than the native species [13,38]. In the present study, the annual average CCM and CCA of *Sonneratia* were 7.7% and 18.4% lower than those of the native species, respectively. The lower leaf CC suggested that the alien *Sonneratia* species would require less energy for biomass construction and that the saved energy could be funneled towards other uses, such as seed production. The lowest CCM and CCA were found in young Sa and Sc plants, respectively, among all six species. These results above on seedlings of less than 3-years old were in agreement with previously reported results on adult and mature mangrove plants [32].

Comparing the same index (CC) of seedlings and adults, it was found that CC of *Sonneratia* species were significantly lower than that of native mangroves in both seedling and adult stages, indicating the invasiveness of *Sonneratia* occurred in early ages and continued to mature adults thus their invasive potential must not be overlooked. More, in both seedlings and adult stages, Sa had the lowest CCM while Sc had the lowest CCA, reflecting that the competitiveness of the former species was on the biomass accumulation while that of Sc was on the leaf blade area expansion.

PEUE has been used to assess the ratio of energetic gains to cost and reflects the specific energy use strategy of plants [39]. A high PEUE is one of the main components of the high RGR [44,45]. The higher PEUE of the alien *Sonneratia* compared to the native mangrove species in the present study indicated that *Sonneratia* could assimilate significantly more carbon per unit of energy, leading to more biomass construction and a higher RGR than the native mangroves throughout the entire year. This phenomenon is another competitive advantage and enhances the invasive potential of *Sonneratia*.

A high PNUE due to high photosynthesis at a low level of leaf nitrogen may provide a competitive advantage in the coexistence of exotic species with native species [41]. A high PNUE is also an indicator of a high efficiency of resource use [46]. However, the PNUE of *Sonneratia* in the present study was not the highest value, likely because N was not a limiting factor in this mangrove swamp. *Sonneratia* was reported to produce large quantities of litter [31,47] and exhibit rapid decomposition [47,48], thereby providing a continuous and rich supply of N for growth. Moreover, the Futian mangrove swamp in the inner part of Shenzhen Bay has a long history of N contamination because it is located in one of the early developed districts in Shenzhen with a high population density, many processing-related industries, and many roads and highways. The N concentration of the surface soils (0–10 cm) in Futian Nature Reserve was 1.27–2.22% [49–51]. Although *Sonneratia* did not have the highest PNUE, it did have the highest P_n_ and A_total_ and the lowest CC among all mangrove species, which gave a competitive advantage to these alien species. Conversely, Ac had the highest PNUE and P_n_ as well as the highest CC, which significantly prevented its growth among the four native mangroves species.

Multiple integrating factors contribute to the ability of an introduced species to grow quickly and become invasive. Obviously, a high photosynthetic rate is the most important factor for fast plant growth. A low CC is also responsible for saving energy for the investment of other competitive strategies. An alien species with high photosynthetic characteristics but low CC would be more invasive than the species which with: low photosynthesis but high CC, or high photosynthesis and high CC, or low photosynthesis and low CC. In the present study, the A_total_ of *Sonneratia* was 32% greater than that of the native species. The higher A_total_ and P_n_ coupled with the lower CC led to a higher PEUE (72%) and a higher RGR (51% in biomass and 119% in height) of *Sonneratia* than the native mangroves, indicating more invasive of this genus. Although the difference in the photosynthetic indices between Sa and Sc was small, the significantly lower CCM of Sa suggested that this alien species was more invasive. Additionally, the P_n_, A_total_ and PEUE of Sa were comparable to those values in the native species and were higher than those of Sc in the winter when the plants grew slowly. These results explain why the introduction of Sa in Shenzhen has been very successful since 1993 but some Sc plants have been found dead in the winter. Thus, more attention should be paid to Sa in the Shenzhen mangrove swamps in future studies. In the present study, the combined uses of the photosynthetic characteristics and energetic cost in four seasons provided a more comprehensive evaluation on the invasive potential of an alien genus, *Sonneratia*, in mangrove swamps in South China. Their invasiveness was attributed to the higher P_n_ and A_total_ coupled with a lower CC resulted in a higher PEUE of *Sonneratia* that allowed them to save more energy and resources for other growth strategies.

## Conclusion

The higher P_n_ and A_total_ coupled with a lower CC resulted in a higher PEUE of *Sonneratia* that allowed the two alien species (Sa and Sc) to save more energy and resources for other growth strategies, thereby enhancing their invasive potential. The higher photosynthetic indices of *Sonneratia* in spring, summer and fall and the lower CC in all four seasons not only indicated their competitiveness in the three growing seasons but also implied the ability of these two species to preserve energy for the winter. More than 20 years of field observations showed that *Sonneratia* gradually adapted to the cold winter and successfully colonized this subtropical mangrove nature reserve in Shenzhen. Among the two *Sonneratia* species, Sa was more competitive in terms of photosynthesis and CC than Sc, indicating that more attention should be paid to the invasive potential of this alien species in future research.

## Supporting Information

S1 Table T-values of the independent samples T-test showing the differences on the photosynthetic characteristics, energetic cost and growth traits between *Sonneratia* and native mangrove groups. (DOCX)
