# Supplementary material for: Are Photosynthetic Characteristics and Energetic Cost Important Invasive Traits for Alien Sonneratia Species in South China?
Source: PLoS One. 2016 Jun 10;11(6):e0157169. doi: 10.1371/journal.pone.0157169 (PMC4902315; doi:10.1371/journal.pone.0157169)
Supplement: S1 Table — (DOCX) [file pone.0157169.s001.docx]

**S1 Table. T-values of the independent samples T-test showing the differences on the photosynthetic characteristics, energetic cost and growth traits between *Sonneratia* and native mangrove groups.**

| **Items** | **T-value**  **In each season (t_Jan_, t_Apr_, t_Jul_, t_Oct_): n_alien_=6, n_native_=12, df= n_alien_+ n_native_-2=16**  **In a year (t_annual_): n_alien_=24, n_native_=48, df= n_alien_+ n_native_-2=70** | | | | |
| --- | --- | --- | --- | --- | --- |
|  | **t_Jan._** | **t_Apr._** | **t_Jul._** | **t_Oct._** | **t_annual_** |
| **P_n_** | -1.58^NS^ | 5.23^***^ | 3.15^**^ | 2.83^**^ | 3.35^**^ |
| **G_s_** | -2.06^*^ | 5.41^***^ | 2.38^*^ | 2.09^*^ | 2.67^**^ |
| **E** | -2.15^*^ | 4.09^***^ | 1.40^NS^ | 1.75^NS^ | 1.87^NS^ |
| **C_i_** | -1.70^NS^ | 0.95^NS^ | 0.19^NS^ | -0.37^NS^ | -0.80^NS^ |
| **A_total_** | -1.58^NS^ | 5.23^***^ | 3.15^**^ | 2.83^**^ | 3.35^**^ |
| **CCM** | -4.74^***^ | -5.58^***^ | -3.40^**^ | -2.53^*^ | -6.11^***^ |
| **CCA** | -0.28^NS^ | -0.75^NS^ | -4.44^***^ | -2.66^*^ | -3.48^**^ |
| **PEUE** | -0.90^NS^ | 2.28^*^ | 6.11^***^ | 3.67^**^ | 4.22^***^ |
| **PNUE** | -1.18^NS^ | 1.75^NS^ | 2.84^*^ | 0.35^NS^ | 1.55^NS^ |
| **RGR_Biomass_** | 4.49^***^ | 0.73^NS^ | 0.24^NS^ | 2.13^*^ | 2.39^*^ |
| **RGR_Height_** | 14.51^***^ | -0.32^NS^ | 0.32^NS^ | 0.92^NS^ | 2.47^*^ |
| **RGR_Diameter_** | -0.86^NS^ | 1.00^NS^ | 1.11^NS^ | 1.93^NS^ | 1.88^NS^ |

Items in the first column were same as Table 2. t_Jan._,t_Apr._, t_Jul._, t_Oct._, and t_annual_ represent the t-values of T-text between *Sonneratia* and native mangroves in winter, spring, summer, fall and annual, respectively. Levels of significance are shown as ^*^ *p ≤*0.05, ^**^ *p ≤*0.01, ^***^ *p ≤*0.001 and NS for not significant at *p ≤*0.05.
